# Pharmacokinetic/pharmacodynamic parameters of teicoplanin for predicting clinical outcome of glycopeptide-susceptible *Enterococcus faecium* bacteraemia

**DOI:** 10.1093/jacamr/dlaf151

**Published:** 2025-08-21

**Authors:** Ryo Yamaguchi, Takehito Yamamoto, Sohei Harada, Mayu Shibuya, Miyuki Mizoguchi, Yoshimi Higurashi, Miho Echizenya, Takeya Tsutsumi, Tappei Takada

**Affiliations:** Department of Pharmacy, The University of Tokyo Hospital, Tokyo, Japan; Department of Pharmacy, The University of Tokyo Hospital, Tokyo, Japan; Department of Microbiology and Infectious Diseases, Toho University School of Medicine, Tokyo, Japan; Department of Infection Control and Prevention, The University of Tokyo Hospital, Tokyo, Japan; Department of Infection Control and Prevention, The University of Tokyo Hospital, Tokyo, Japan; Department of Infection Control and Prevention, The University of Tokyo Hospital, Tokyo, Japan; Department of Pharmacy, The University of Tokyo Hospital, Tokyo, Japan; Department of Infection Control and Prevention, The University of Tokyo Hospital, Tokyo, Japan; Department of Infectious Diseases, The University of Tokyo Hospital, Tokyo, Japan; Department of Pharmacy, The University of Tokyo Hospital, Tokyo, Japan

## Abstract

**Objectives:**

The objective of this study was to determine the pharmacokinetic/pharmacodynamic parameters of teicoplanin associated with optimal outcomes in glycopeptide-susceptible *Enterococcus faecium* (GSEF) bacteraemia.

**Patients and methods:**

We conducted a retrospective review of GSEF bacteraemia cases treated with teicoplanin between 1 April 2009 and 30 May 2023. Total area under the concentration–time curve over 24 h (AUC_24_) was calculated using a Bayesian approach. The free AUC_24_ (fAUC_24_) was estimated based on patient serum albumin levels. MICs were determined using the gradient diffusion method (Etest), and the fAUC_24_/MIC_Etest_ ratio was calculated. The primary outcome was treatment failure, defined as a composite of (i) 30-day all-cause mortality and (ii) microbiological failure, defined as persistent bacteraemia (a positive follow-up blood culture obtained >72 h after initiation of appropriate therapy). Classification and regression tree analysis (CART) was employed to identify the optimal teicoplanin fAUC_24_/MIC_Etest_ value associated with treatment failure.

**Results:**

A total of 76 patients were included. Treatment failure occurred in 18 patients (23.7%). A CART-derived teicoplanin fAUC_24_/MIC_Etest_ ≥ 462 was significantly associated with reduced treatment failure (*P* = 0.002). Multivariable regression analysis revealed that achievement of an fAUC_24_/MIC_Etest_ ≥ 462 was an independent predictor significantly associated with reduced treatment failure (OR, 0.099; 95% CI, 0.005–0.562; *P* = 0.032).

**Conclusions:**

An fAUC_24_/MIC_Etest_ ≥ 462 was associated with a reduction in treatment failure in GSEF bacteraemia. Further studies are necessary to establish optimal pharmacokinetic/pharmacodynamic targets for GSEF bacteraemia.

## Introduction

Enterococci are commensal bacteria residing in the intestinal tract, yet they can cause serious infections.^1^ Mortality rates associated with enterococcal bacteraemia have been reported to range between 20% and 40%.^2–4^ The European Antimicrobial Resistance Surveillance System (EARSS) database has documented a continuous increase in *Enterococcus faecium* bacteraemia cases, with vancomycin-resistant *E. faecium* (VRE) emerging as a major global concern.^5,6^ While the prevalence of VRE remains relatively low in Japan,^7^ this regional heterogeneity underscores the critical need to optimize treatment strategies for glycopeptide-susceptible *E. faecium* (GSEF) infections to preserve the efficacy of available agents such as teicoplanin.

Teicoplanin, a glycopeptide antibiotic, is widely used in Europe and the Asia-Pacific region, including Japan, due to its superior *in vitro* activity against enterococci compared to vancomycin and its longer half-life, which allows once-daily dosing.^8^ Our previous research evaluated the effectiveness and safety of teicoplanin versus vancomycin in the treatment of GSEF bacteraemia.^9^ The results demonstrated that teicoplanin was non-inferior to vancomycin for GSEF bacteraemia treatment, with a significantly lower incidence of acute kidney injury (AKI) associated with teicoplanin. Based on these findings, teicoplanin may be considered a viable alternative to vancomycin in the treatment of GSEF bacteraemia. However, the relationship between the pharmacokinetic/pharmacodynamic (PK/PD) parameters of teicoplanin and its therapeutic effectiveness in GSEF bacteraemia has yet to be fully elucidated.

The PK/PD parameter most commonly associated with the clinical efficacy of teicoplanin against MRSA is the ratio of the AUC to the MIC, referred to as AUC/MIC.^10,11^ The target AUC/MIC ratio for teicoplanin against MRSA is approximately 600–900, and achieving this target is known to improve therapeutic outcomes.^12,13^ Furthermore, teicoplanin is a highly protein-bound drug (approximately 90%), and it is the unbound (free) fraction that exerts antimicrobial activity. In critically ill patients, conditions such as hypoalbuminaemia can alter protein binding, potentially affecting the free drug concentration and therapeutic efficacy. Therefore, evaluating the free AUC/MIC (fAUC/MIC) ratio is considered more pharmacologically relevant than using the total concentration. However, the relationship between the fAUC/MIC ratio and the therapeutic effectiveness of teicoplanin in GSEF bacteraemia, along with the optimal target values, remains unclear. Consequently, the current dosing regimen for teicoplanin in GSEF bacteraemia is extrapolated from the regimen used for MRSA. Given that different bacterial strains may have distinct PK/PD targets even when treated with the same antibiotic, the optimal PK/PD parameters of teicoplanin for GSEF bacteraemia differ from those for MRSA, necessitating further investigation to determine a more appropriate fAUC/MIC ratio.^14^

This study aimed to elucidate the relationship between the fAUC/MIC ratio of teicoplanin and its therapeutic effectiveness for GSEF bacteraemia and to identify the optimal target fAUC/MIC ratio.

## Patients and methods

### Study design and setting

We conducted a retrospective, single-centre, observational cohort study at The University of Tokyo Hospital between 1 April 2009 and 30 May 2023. The University of Tokyo’s ethics review board approved the study protocol (approval no. 2023138NI) and waived the requirement for written informed consent. The study was conducted in accordance with the ethical standards of the 1964 Declaration of Helsinki and its subsequent amendments. Patients with at least one GSEF-positive blood culture who received teicoplanin during the study period were deemed eligible. The exclusion criteria were as follows: lack of storage of microbiological samples, lack of teicoplanin trough concentration (*C*_min_), age under 18 years, renal replacement therapy or initiation of teicoplanin treatment more than 72 h after infection. GSEF bacteraemia was defined as one or more blood cultures positive for *E. faecium* resistant to ampicillin, and susceptible to teicoplanin and vancomycin, accompanied by signs or symptoms of infection consistent with suspected infection sites, as established in previous studies.^9,15,16^ Polymicrobial infections were included in the analysis. For patients with a history of multiple treatments for GSEF bacteraemia, only the first treatment was considered.

### Data collection

We collected demographic and clinical data from patient medical records, including age, sex, height, weight, infection type (community- or hospital-acquired), ICU stay, comorbidities, Charlson Comorbidity Index (CCI), Pitt bacteraemia score and laboratory data such as serum creatinine (sCr), serum albumin (ALB), haemoglobin, total bilirubin (T-Bil), blood urea nitrogen (BUN), C-reactive protein, white blood cell count, absolute neutrophil count (ANC) and platelet count. These data were recorded on or immediately prior to the day the first GSEF-positive blood culture was obtained. Community-acquired infections were defined as those when the first GSEF-positive blood culture was obtained ≤48 h after hospitalization, while hospital-acquired infections were defined as those when the first positive culture occurred >48 h after admission. The estimated glomerular filtration rate (eGFR) was calculated using the formula recommended by the Japanese Society of Nephrology.^17^ Neutropenia was defined as an ANC < 500 cells/mm^3^.^18^ Immunocompromised patients were those who had received cancer chemotherapy, radiation therapy or immunosuppressive agents, including calcineurin inhibitors or corticosteroids, within the 30 days preceding the first GSEF-positive blood culture.^19^ The sources of bacteraemia were determined based on microbiological and clinical criteria.^16^ Therapy duration was defined as the period of teicoplanin administration. Source control, such as endoscopic retrograde cholangiopancreatography, percutaneous transhepatic biliary drainage or central venous catheter removal, was defined as any incision, drainage, or debridement performed during teicoplanin therapy. Appropriate antimicrobial therapy was defined as the intravenous administration of antimicrobial agents to which all isolated pathogens were susceptible, within 24 h of obtaining the positive blood culture.^2,20^ Co-administered antibiotics other than teicoplanin were reviewed to assess the appropriateness of antimicrobial therapy.

### Microbiological data

Blood culture specimens were processed using the BACTEC FX system (Becton Dickinson Microbiology Systems, Sparks, MD, USA). Species identification of bacterial isolates recovered from blood cultures was performed using the MALDI Biotyper 1.0 (Bruker Daltonics, Bremen, Germany). Antimicrobial susceptibilities to ampicillin and teicoplanin were tested using the MicroScan WalkAway system (Beckman Coulter Japan, Tokyo, Japan) and interpreted according to the Clinical and Laboratory Standards Institute (M100-S26) breakpoints.^21^ Polymicrobial bacteraemia was defined as cases where one or more microorganisms, in addition to *E. faecium*, were isolated from the same blood culture and were not classified as contaminants according to the CDC criteria.^16^

For this study, teicoplanin MIC determinations were performed on archived isolates using a commercial automated broth microdilution method with the MicroScan WalkAway-96 Plus system (MIC_WalkAway_) (Beckman Coulter) and gradient diffusion method (MIC_Etest_) (Etest, bioMérieux) following the manufacturer’s instructions. Due to a change in the MIC measurement range during the study period, all isolates were remeasured using the MicroScan WalkAway-96 Plus system for consistency. The MIC_WalkAway_ measurement range was 2, 4, 8, and 16 mg/L. The MIC_Etest_ results were reviewed and confirmed independently by three clinical microbiologists who were blinded to the clinical outcomes of the patients from whom the isolates were obtained. In cases where disagreement occurred among all three operators, the MIC was retested and determined by the representative operator, taking the initial results into consideration.

### Teicoplanin dosing and PD data

The primary PK/PD parameter for this study was the ratio of the estimated 24-h free-drug area under the curve to the MIC (fAUC_24_/MIC_Etest_). The estimation of fAUC_24_ was based on total *C*_min_ measured during the maintenance phase of therapy (at least 3 days after treatment initiation). Our institutional dosing protocol recommends a loading dose of 8–10 mg/kg administered three times approximately every 12 h within the first 48 h for patients with normal renal function, followed by a maintenance dose of 6 mg/kg once daily from Day 3. This loading regimen is intended to rapidly achieve therapeutic concentrations, allowing *C*_min_ values measured during the maintenance phase to serve as a reliable surrogate for drug exposure. Teicoplanin doses were routinely proposed by a pharmacist using therapeutic drug monitoring (TDM) with a target *C*_min_ for teicoplanin set at 15–30 mg/L in line with guideline recommendations,^22^ though actual dosing was determined at the discretion of the attending physician.

The estimation of fAUC_24_ involved a multi-step process. First, the total drug AUC over a 24-h interval during the maintenance phase (AUC_24_) was calculated for each patient. This was based on individual total clearance (CL_total_) estimated from the observed *C*_min_ through Bayesian analysis. This analysis employed a previously developed population PK (PopPK) model for Japanese patients constructed by Nakayama et al.^23^ This PopPK model was based on data from 120 Japanese adult inpatients with MRSA infections and incorporates creatinine clearance and body weight as covariates, making it applicable to our study population. The reported inter-individual variabilities (CV%) for the main parameters were 22.1% for clearance (CL) and 26.7% for volume of distribution (*V*), with a residual variability of 15.6%. Based on the estimated CL_total_ for each patient, the AUC_24_ was calculated as AUC_24_ (mg·h/L) = daily maintenance dose (mg)/CL_total_ (L/h).^13,24^ Second, to account for the impact of hypoalbuminaemia, a patient-specific unbound fraction (fu) was estimated based on a validated model developed specifically for teicoplanin in a Japanese patient population by Yano et al.^25^ The model predicts the fu using the patient’s ALB concentration with the following formula:


fu=1/(1+1.78×ALB)

where ALB is expressed in g/dL.

Finally, the free AUC_24_ (fAUC_24_) was estimated by multiplying the total AUC_24_ by fu. The resulting fAUC_24_/MIC_Etest_ ratio was used for all subsequent PK/PD analyses.

PK parameters for each patient were determined using the TDM software, BMs-Pod (ver 8.06, https://bmspod.web.fc2.com/).^26,27^

### Clinical outcomes

The primary outcome, treatment failure, was defined as a composite of (i) 30-day all-cause mortality or (ii) microbiological failure, defined as persistent bacteraemia (a positive follow-up blood culture obtained >72 h after initiation of appropriate therapy) in patients with at least one follow-up blood culture).^28,29^ In patients without follow-up blood cultures, microbiological failure was not assessed, and treatment failure was determined solely based on 30-day all-cause mortality. Patients who did not meet the criteria for treatment failure were classified as having treatment success. Secondary outcomes included 90-day all-cause mortality (death within 90 days of the first positive GSEF blood culture), in-hospital mortality (death occurring during hospitalization), relapse (recurrence of GSEF bacteraemia after negative blood cultures following GSEF treatment) and readmission within 90 days of the first positive GSEF blood culture. Relapse was defined as recurrence of GSEF bacteraemia after documented microbiological clearance, confirmed by at least one negative follow-up blood culture. All clinical outcomes and PK parameter estimation (including AUC calculation) were conducted by an infectious disease pharmacist with access to treatment details and patient clinical courses. MIC measurements were performed independently by separate investigators and were integrated only after clinical outcome evaluation was completed. Safety outcomes included (i) incidence of AKI, diagnosed according to the KDIGO criteria (defined as 1.5× increase in sCr from baseline within 7 days or ≥0.3 mg/dL increase within 48 h of baseline following teicoplanin initiation)^30^; (ii) incidence of liver injury, defined as AST ≥ 90 U/L or ALT ≥ 126 U/L (≥3 times the upper limit during teicoplanin treatment)^31^; and (iii) other adverse events associated with teicoplanin use for GSEF bacteraemia.^15^

### Statistical analysis

Continuous variables, expressed as medians with IQRs, were compared between the treatment success and treatment failure groups using the Mann–Whitney *U*-test. Categorical variables were compared between the two groups using the χ² test or Fisher’s exact test, as appropriate. To identify predictors of treatment failure, several steps were taken for the logistic regression analysis. First, classification and regression tree (CART) analysis was used to identify the optimal cut-off value for the fAUC_24_/MIC_Etest_ associated with treatment failure, according to the previous vancomycin PK/PD study.^32^ This created a dichotomous variable for the PK/PD parameter. Next, a multivariable logistic regression model was constructed to assess the independent effect of the fAUC_24_/MIC_Etest_ on treatment failure. To ensure the model was clinically robust and to adjust for key prognostic factors, we included several covariates based on their established clinical importance in severe infections, as guided by previous literature.^33–35^ Specifically, the CCI, immunosuppressed state and Pitt bacteraemia score were forced into the model as adjustment variables. Multicollinearity was assessed by calculating variance inflation factors (VIFs), with a VIF > 10 indicating collinearity. In cases of collinearity, only one variable was included in the analysis. Model calibration was evaluated with the Hosmer–Lemeshow goodness-of-fit test, and discrimination was assessed using the area under the receiver operating characteristic curve. All analyses were performed using R version 4.3.1 (R Foundation for Statistical Computing, Vienna, Austria) and JMP Pro 18.0.1 (SAS Institute Inc., Cary, NC, USA). A two-sided *P* value < 0.05 was considered statistically significant.

## Results

### Characteristics of patients

**Figure 1.**
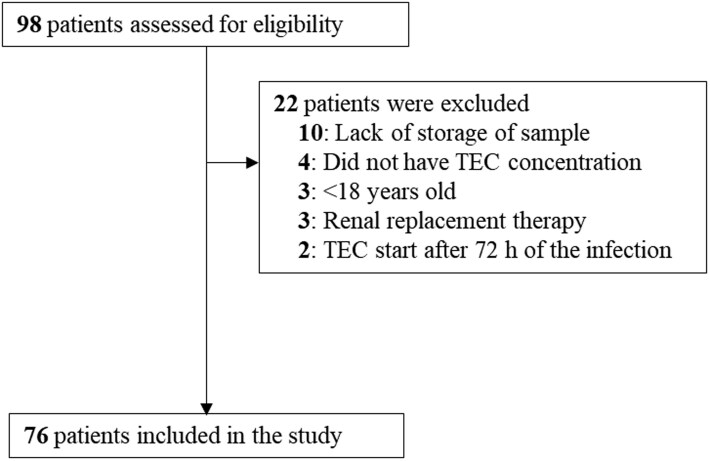
Flowchart of patient selection.

We identified 98 patients with *E. faecium* bacteraemia who received teicoplanin treatment, of whom 76 were included in the final analysis (Figure 1). The baseline characteristics of the patients are summarized in Table 1. The median ages were 61.5 years (IQR, 53.3–74.5) in the treatment success group and 56.5 years (IQR, 52.0–66.0) in the treatment failure group. Mechanical ventilation was used more frequently in the treatment failure group than in the treatment success group (27.8% versus 3.4%, *P* = 0.007). More than half of the patients in both groups were in an immunosuppressed state. Appropriate antimicrobial therapy and source control were implemented in over 80% of cases in both groups. Because the distribution of polymicrobial infections was balanced between the groups and appropriate therapy was achieved in more than 80% of cases in both groups, polymicrobial infection was not included as a variable in the multivariable regression analysis.

**Table 1.** Baseline characteristics of patients

Characteristic	Overall (*n* = 76)	Treatment success (*n* = 58)	Treatment failure (*n* = 18)	*P* value
Male sex	51 (67.1)	40 (69.0)	11 (61.1)	0.574
Age (years)	61.0 (52.0–73.0)	61.5 (53.3–74.5)	56.5 (52.0–66.0)	0.416
Height (cm)	163.1 (156.6–170.8)	162.8 (156.1–168.0)	164.8 (160.3–172.8)	0.356
Weight (kg)	60.9 (53.4–67.0)	60.3 (48.5–66.7)	63.0 (56.8–71.6)	0.231
BMI	22.3 (19.7–25.3)	22.1 (19.7–25.1)	22.9 (20.3–26.5)	0.282
ICU stay	22 (28.9)	15 (25.9)	7 (38.9)	0.373
Mechanical ventilation	7 (9.2)	2 (3.4)	5 (27.8)	0.007
Hospital-acquired infection^a^	67 (88.2)	49 (84.5)	18 (100.0)	0.105
Charlson Comorbidity Index	4.0 (3.0–6.0)	4.0 (3.0–6.0)	3.0 (2.3–5.0)	0.440
Pitt bacteraemia score	1.0 (0.0–2.2)	1.0 (0–2.0)	1.5 (0–4.8)	0.313
Hypertension	20 (26.3)	16 (27.6)	4 (22.2)	0.766
Chemotherapy	14 (18.4)	12 (20.7)	2 (11.1)	0.497
COPD	4 (5.3)	4 (6.9)	0 (0)	0.567
WBC count (×10^3^ cells/mm^3^)	7.6 (5.0–12.0)	8.1 (5.8–12.3)	5.5 (4.2–11.8)	0.224
Neutrophil count (×10^3^ cells/mm^3^)	6.0 (3.8–9.8)	7.1 (4.2–9.7)	3.9 (2.3–8.9)	0.079
Neutropenia^b^	9 (11.8)	6 (10.3)	3 (16.7)	0.435
Haemoglobin (g/dL)	8.8 (7.6–10.2)	9.4 (8.2–10.5)	8.2 (6.4–8.5)	0.002
Platelet count (×10^3^/mm^3^)	10.6 (5.4–21.9)	12.5 (6.0–23.9)	6.3 (3.1–13.9)	0.036
BUN	21.0 (14.9–37.5)	17.7 (13.9–30.7)	34.7 (20.0–50.8)	0.010
Serum creatinine (mg/dL)	1.0 (0.7–1.3)	0.9 (0.7–1.3)	1.1 (0.8–1.6)	0.149
eGFR (mL/min/1.73 m^2^)	58.5 (40.0–86.4)	62.4 (42.5–86.5)	47.8 (38.0–60.6)	0.160
Serum albumin level (g/dL)	2.9 (2.6–3.2)	2.8 (2.6–3.2)	3.0 (2.8–3.3)	0.342
Total bilirubin (mg/dL)	1.6 (0.9–3.2)	1.6 (0.80–2.48)	3.7 (1.5–8.4)	0.004
CRP (mg/dL)	5.8 (2.5–8.9)	5.7 (2.6–9.3)	6.6 (2.7–8.5)	0.951
Immunosuppressed state^c^	46 (60.5)	32 (55.2)	14 (77.8)	0.104
Source of infection				
Hepatobiliary	32 (42.1)	27 (46.6)	5 (27.8)	0.571
Intravascular catheter	14 (18.4)	9 (15.5)	5 (27.8)	
Febrile neutropenia	3 (3.9)	3 (5.2)	0 (0)	
Intra-abdominal	16 (21.1)	11 (19.0)	5 (27.8)	
Surgical site	9 (11.8)	6 (10.3)	3 (16.7)	
Others^d^	2 (2.6)	2 (3.4)	0 (0)	
Concomitant antibiotics				
PIP/TZP	43 (56.6)	35 (60.3)	8 (44.4)	0.575
MEM	21 (27.6)	14 (24.1)	7 (38.9)	
DOR	3 (3.9)	2 (3.4)	1 (5.6)	
FEP	2 (2.6)	2 (3.4)	0 (0)	
Others^e^	7 (9.2)	5 (8.6)	2 (11.1)	
Polymicrobial infections	24 (31.6)	20 (34.5)	4 (22.2)	0.396
Appropriateness of therapy	67 (88.2)	49 (84.5)	18 (100)	0.105
Duration of therapy	19.5 (14.0–30.0)	17.0 (14.0–26.8)	29.5 (14.0–50.3)	0.055
Source control^f^	62 (81.6)	47 (81.0)	15 (83.3)	1.000
Length of hospital stay	60.5 (37.8–104.2)	51.0 (36.3–94.8)	109.5 (65.3–181.5)	0.010

Data are presented as medians (IQRs) for continuous variables and *n* (%) for categorical variables unless otherwise stated.

BMI, body mass index; BUN, blood urea nitrogen; COPD, chronic obstructive pulmonary disease; WBC, white blood cell; eGFR, estimated glomerular filtration rate; CRP, C-reactive protein; PIP/TZP, piperacillin/tazobactam; MEM, meropenem; DOR, doripenem; FEP, cefepime.

^a^Bacteraemia after 48 h of hospitalization.

^b^Neutropenia was defined as a neutrophil count of <500/mm^3^.

^c^Use of chemotherapy and/or immunosuppressive drugs (corticosteroids, cyclosporin A/tacrolimus) within 1 month before bacteraemia.

^d^Respiratory (1) and urinary tract infection (1).

^e^Ciprofloxacin (1), cefmetazole (1), ceftriaxone (1), imipenem/cilastatin (1), minocycline plus ceftazidime (1), cefepime plus clindamycin (1) and no concomitant antibiotic (1).

^f^Incision, drainage and debridement (including endoscopic retrograde cholangiopancreatography, percutaneous transhepatic biliary drainage and removal of the central venous catheter).

### PK/PD analysis and target identification

The primary outcome, treatment failure, occurred in 18 of 76 patients (23.7%). Follow-up blood cultures were obtained in 63 of 76 cases (83%). The overall 30-day mortality rate was 7.9% (6/76). To investigate the relationship between drug exposure and primary outcome, we first analysed the MIC distribution and then the teicoplanin PK/PD parameters. The MIC of each isolate was determined by Etest, as all MIC_WalkAway_ values were ≤2 mg/L. The MIC_Etest_ values for the 76 isolates ranged from 0.023 to 1 mg/L. The MIC_Etest_ retests were performed on eight isolates according to the study protocol, and all retest results were within a 2-fold range of the initial values. As shown in Table 2, there was a significant difference in the MIC_Etest_ distribution between the treatment success and failure groups [median 0.25 mg/L (IQR, 0.13–0.50) versus 0.50 mg/L (IQR, 0.35–0.50); *P* = 0.008].

**Table 2.** Teicoplanin PK/PD parameters

PK/PD parameters	Treatment success (*n* = 58)	Treatment failure (*n* = 18)	*P* value
MIC_Etest_	0.25 (0.13–0.50)	0.50 (0.35–0.50)	0.008
*C*_min_ (mg/L)	18.3 (15.0–23.1)	18.4 (9.70–23.1)	0.783
*C*_min_/MIC_Etest_	72.1 (35.9–142.4)	42.0 (22.2–67.2)	0.013
fAUC_24_ (mg·h/L)	96.9 (81.2–119.6)	96.9 (83.4–113.9)	0.869
fAUC_24_/MIC_Etest_	412.3 (204.6–652.3)	245.3 (167.6–372.5)	0.021
fAUC_24_/MIC_Etest_ ≥ 462	26 (44.8)	1 (5.6)	0.002

Data are presented as medians (IQRs) for continuous variables and *n* (%) for categorical variables unless otherwise stated.

PK, pharmacokinetics; PD, pharmacodynamics; MIC, minimum inhibitory concentration; fAUC, free area under the curve.

**Figure 2.**
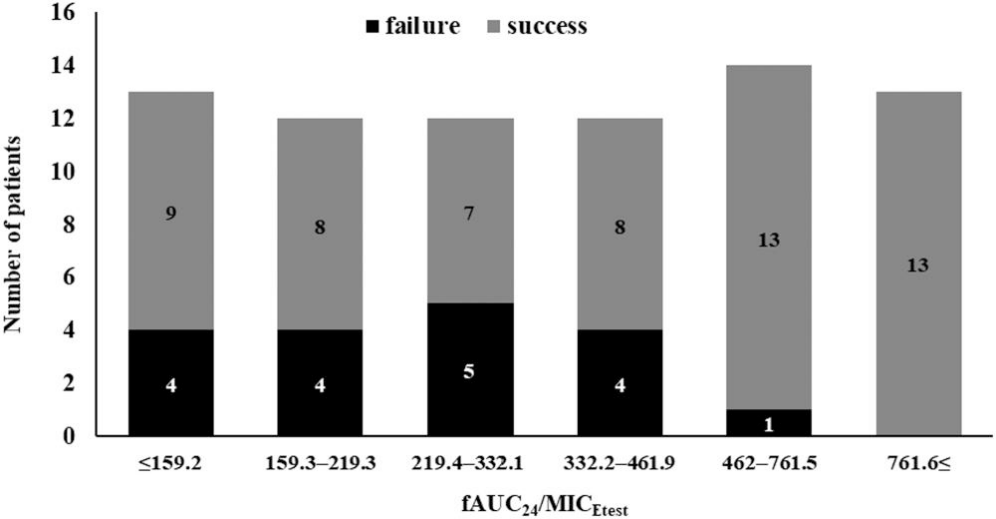
Proportion of treatment failure according to fAUC_24_/MIC_Etest_ values. The bar graph shows the number of patients with treatment success and treatment failure stratified by fAUC_24_/MIC_Etest_ values. fAUC, free area under the curve.

Next, we compared several teicoplanin PK/PD parameters between the groups (Table 2). The PK analysis for our 76 patients yielded substantial inter-individual variability (CV%) for clearance (39.2%) and steady-state volume of distribution (43.8%), while variability was low for the distribution rate parameters, α (4.7%) and K12 (0.1%). The median fAUC_24_/MIC_Etest_ was found to be significantly higher in the treatment success group than in the treatment failure group [412.3 (IQR, 204.6–652.3) versus 245.3 (IQR, 167.6–372.5); *P* = 0.021]. In contrast, there were no significant differences in total *C*_min_ or fAUC_24_ values alone. Based on this finding, we used CART analysis to determine the optimal fAUC_24_/MIC_Etest_ cut-off value for predicting treatment failure, which was identified as 462. Figure 2 shows the distribution of treatment outcomes across different fAUC_24_/MIC_Etest_ ranges. It demonstrates a marked reduction in treatment–failure ratio among patients with an fAUC_24_/MIC_Etest_ ≥ 462.

### Clinical outcomes according to PK/PD target and toxicity analysis

To evaluate the clinical relevance of this PK/PD target, we stratified patients by this cut-off and compared their outcomes (Table 3). Patients who achieved the target (fAUC_24_/MIC_Etest_ ≥ 462) had a significantly lower incidence of treatment failure compared to those who did not (3.7% versus 34.7%; *P* = 0.002).

**Table 3.** Clinical outcomes according to PK/PD exposure

Outcomes	fAUC_24_/MIC_Etest_ < 462 (*n* = 49)	fAUC_24_/MIC_Etest_ ≥ 462 (*n* = 27)	*P* value
Treatment failure	17 (34.7)	1 (3.7)	0.002
30-day mortality	6 (12.2)	0 (0.0)	0.083
90-day mortality	17 (34.7)	3 (11.1)	0.031
In-hospital mortality	12 (24.5)	2 (7.4)	0.120
Relapse	7 (14.3)	3 (11.1)	1.000
Readmission within 90 days after bacteraemia	22 (44.9)	8 (29.6)	0.227

Data are presented as *n* (%) for categorical variables.

fAUC, free area under the curve; MIC, minimum inhibitory concentration; ADR, adverse drug reaction.

To explore the relationship between drug exposure and toxicity, fAUC_24_ and *C*_min_ values were compared between patients with and without adverse events. There were no significant differences in median fAUC_24_ or *C*_min_ values for patients who experienced nephrotoxicity (*n* = 6) compared to those who did not (fAUC_24_: 86.2 versus 99.4 mg·h/L, *P* = 0.184; *C*_min_: 17.1 versus 18.4 mg/L, *P* = 0.470). Similarly, no significant association was found between drug exposure and the incidence of liver injury or other adverse drug reactions.

### Predictors of treatment failure

The results of the multivariable logistic regression analysis are shown in Table 4. After adjusting for the CCI, immunosuppressed state and Pitt bacteraemia score, achieving an fAUC_24_/MIC_Etest_ ≥ 462 remained an independent predictor of reduced treatment failure (OR, 0.099; 95% CI, 0.005–0.562; *P* = 0.032). In this model, the other covariates were not significantly associated with the outcome.

**Table 4.** Multivariable analysis of teicoplanin treatment failure

Variables	Univariable		Multivariable	
	Odds ratio (95% CI)	*P* value	Odds ratio (95% CI)	*P* value
Teicoplanin fAUC_24_/MIC_Etest_ ≥ 462	0.072 (0.004–0.389)	0.013	0.099 (0.005–0.562)	0.032
Charlson Comorbidity Index	0.936 (0.744–1.142)	0.540	0.935 (0.709–1.184)	0.602
Immunosuppressed state	2.844 (0.895–10.979)	0.095	2.603 (0.709–11.548)	0.170
Pitt bacteraemia score	1.272 (1.019–1.611)	0.035	1.227 (0.975–1.580)	0.089

fAUC, free area under the curve; MIC, minimum inhibitory concentration.

All variables in the final model had VIFs below 2, indicating no significant multicollinearity. Model calibration was assessed using the Hosmer–Lemeshow goodness-of-fit test, which indicated a good fit (*P* = 0.149). Discrimination, evaluated by the area under the receiver operating characteristic curve, was 0.792, indicating acceptable model performance.

## Discussion

This study aimed to determine the teicoplanin PK/PD target associated with clinical outcomes in patients with GSEF bacteraemia. We found that a teicoplanin fAUC_24_/MIC_Etest_ value of ≥462 was an independent predictor of reduced treatment failure, after adjusting for key clinical prognostic factors (Table 4). To the best of our knowledge, this is the first study to identify a specific fAUC_24_/MIC_Etest_ target for teicoplanin in this patient population.

Our analysis revealed that while total *C*_min_ and fAUC_24_ values did not differ between the treatment success and failure groups, the PK/PD indices incorporating MIC, namely fAUC_24_/MIC_Etest_ and *C*_min_/MIC_Etest_, were significantly different (Table 2). This underscores the critical role of MIC in determining therapeutic outcomes for GSEF bacteraemia. The median MIC_Etest_ in the treatment failure group was double that of the success group (0.50 versus 0.25 mg/L), highlighting that even small variations in MIC can substantially impact the achievement of PK/PD targets. The MIC values in our cohort were relatively low compared to some reports from other regions,^36^ suggesting that the PK/PD targets identified here may need to be validated in populations with different MIC distributions. Our previous report demonstrated that teicoplanin was non-inferior to vancomycin in efficacy, with a treatment success ratio of 64.9% in patients with GSEF bacteraemia; however, regions with different MIC distributions may yield different outcomes.^9^ Studies comparing antimicrobial susceptibility testing methods for enterococci, including Etest, broth microdilution, disk diffusion and automated methods, have shown discrepancies in teicoplanin MIC values across methods.^37,38^ Notably, these studies primarily focused on VRE, offering limited data applicable to GSEF. To ensure the reliability of MIC_Etest_ values in this study, three clinical microbiologists were involved, with retesting conducted in cases of disagreement to minimize inter-operator variability. This approach enhances the reliability of the MIC_Etest_ results. Future studies should further evaluate the reliability of teicoplanin MIC testing methods specifically for GSEF.

A key aspect of our study was the estimation of free drug concentrations. Teicoplanin is highly protein bound, and conditions such as hypoalbuminaemia, common in critically ill patients, can alter this binding. By calculating a patient-specific fu, we aimed to provide a more pharmacologically relevant assessment of drug exposure. While this estimation relies on a theoretical formula and a literature-derived fu, this approach is considered a valid alternative when direct measurement of free concentrations is not feasible.

The accuracy of AUC estimation itself, derived from a single trough concentration via a Bayesian approach, is another important consideration. In this study, we chose a two-compartment model by Nakayama et al., which is well-validated in Japanese patients. Although estimating distribution phase parameters from a single trough point has inherent limitations, our analysis of the individual parameters provided some reassurance. As shown in the Results, while the variability for distribution rate parameters was low, the substantial inter-individual variability for clearance (CV = 39.2%) and volume of distribution (*V*_dss_, CV = 43.8%) suggests that the model robustly estimated these key parameters for each patient. Nevertheless, this single-point estimation method does not capture the impact of drug exposure during the critical initial phase of treatment and remains a limitation of our study. Future prospective studies with serial sampling during initial phase are warranted.

We discuss two perspectives on how to translate the findings of this study into clinical practice. First, we consider the importance of precise MIC determination. This study demonstrated the critical role of MIC values below the CLSI susceptibility breakpoint of 2 mg/L.^21^ However, Etest is not routinely performed in many clinical settings. Our findings suggest that even small variations in MIC can substantially impact the achievement of PK/PD targets. Clinically, if a patient’s response is suboptimal despite adequate *C*_min_ levels, it is essential to consider the possibility of a higher-than-expected MIC. In such cases, confirming the precise MIC value with Etest and subsequently adjusting the dosage to increase fAUC or switching to an alternative agent may be necessary.

Second, we consider the utility of *C*_min_ as a surrogate marker. Since software for assessing fAUC is not widely available, *C*_min_ remains a practical alternative. In our study, the median *C*_min_ in both groups was approximately 18 mg/L, aligning with the recommended therapeutic range of 15–30 mg/L for MRSA.^22,39^ Given that the majority of isolates in our cohort (84.2%) had an MIC ≤ 0.5 mg/L, a target *C*_min_ of 15–30 mg/L appears effective for treating most GSEF infections in regions with similar MIC distributions. This target *C*_min_ likely ensures that the fAUC_24_/MIC_Etest_ target of ≥462 is achieved for these low-MIC isolates.

Regarding safety, our analysis showed no significant association between higher drug exposure (fAUC_24_ or *C*_min_) and the incidence of nephrotoxicity or other adverse events. This suggests that optimizing dosing to achieve the efficacy target of fAUC_24_/MIC_Etest_ ≥ 462 is unlikely to increase the risk of toxicity, a finding consistent with previous meta-analyses on teicoplanin safety.^22,40^

Despite the important clinical implications of this study, several limitations should be discussed. First, this was a retrospective analysis. While the multivariable analysis adjusted for patient backgrounds between the treatment success and treatment failure groups, unmeasured confounders that may have influenced treatment failure cannot be excluded. Known risk factors associated with clinical outcomes of teicoplanin therapy were systematically collected. Second, microbiological failure could not be assessed in all patients, which may have led to misclassification of the primary outcome. Third, this single-centre study was conducted at a university hospital, limiting the generalizability of the findings, particularly since over half of the participants were immunosuppressed. Fourth, the relatively small sample size, particularly the number of treatment failure cases, may have limited our ability to identify other significant predictors of the outcome.

Despite these limitations, this is the first study to identify a target fAUC_24_/MIC_Etest_ value for teicoplanin in GSEF bacteraemia. The findings provide evidence supporting the importance of PK-/PD-based regimens in the treatment of GSEF bacteraemia.

In conclusion, this study demonstrated that a teicoplanin fAUC_24_/MIC_Etest_ value of ≥462 was associated with reduced treatment failure in patients with GSEF bacteraemia, without increasing adverse drug events. Larger prospective studies are necessary to confirm these PK/PD targets for optimizing clinical outcomes and safety.
